# Horses Solve Visible but Not Invisible Displacement Tasks in an Object Permanence Paradigm

**DOI:** 10.3389/fpsyg.2020.562989

**Published:** 2020-09-29

**Authors:** Miléna Trösch, Anna Flamand, Manon Chasles, Raymond Nowak, Ludovic Calandreau, Léa Lansade

**Affiliations:** UMR Physiologie de la Reproduction et des Comportement (PRC), Institut National de Recherche pour l’Agriculture, l’Alimentation et l’Environnement (INRAE), Centre National de la Recherche Scientifique (CNRS), Institut Français du Cheval et de l’Equitation (IFCE), Université de Tours, Nouzilly, France

**Keywords:** *Equus caballus*, object permanence, transposition task, secondary representation, invisible displacement, cognition

## Abstract

A key question in the field of animal cognition is how animals comprehend their physical world. Object permanence is one of the fundamental features of physical cognition. It is the ability to reason about hidden objects and to mentally reconstruct their invisible displacements. This cognitive skill has been studied in a wide range of species but never directly in the horse (*Equus caballus*). In this study, we therefore assessed the understanding of visible and invisible displacements in adult Welsh mares in two complementary experiments, using different horses. In experiment 1, visible displacement was investigated using two tasks adapted from the Uzgiris and Hunt scale 1. Invisible displacement was assessed using a transposition task, in which food was first hidden in one of two containers and the location of the containers was then switched. In experiment 2, we further investigated horses’ understanding of visible and invisible displacements using an easier procedure designed to avoid potentially confounding factors. In both experiments, horses successfully completed the tasks involving visible displacement with two or three possible hiding places. However, in both experiments, horses failed the transposition tasks, suggesting that they may not be able to track the displacement of an object that is not directly perceived (i.e., invisible displacement). These results bring new insights into object permanence in horses and how they represent their physical world.

## Introduction

A key question in the field of animal cognition is how animals comprehend their physical world. Object permanence is one of the fundamental features of physical cognition. It is the ability to consider objects as independent entities that continue to exist even when they are out of sight (Piaget, 1954). It allows organisms to retrieve hidden objects or to reason about the unseen displacement of objects or conspecifics (Piaget, 1954; Shettleworth, 2009). Hence, object permanence seems important for daily survival of animals, as it may be necessary for key activities such as retrieving food in food-storing species but also hunting prey, avoiding predators, or keeping track of conspecifics in social animals (de Blois et al., 1998; Zucca et al., 2007; Auersperg et al., 2014; Jaakkola, 2014). Better knowledge of these cognitive abilities in domestic animals will help improving their housing and management. Indeed, current husbandry conditions usually involve numerous opaque barriers blocking animals’ vision of their surroundings (e.g., walls of stalls or of inside riding areas, transportation vehicles, etc.; Nawroth et al., 2019). A limited understanding of object permanence might thus induce fear reactions, the major cause of horse-related accidents (*Equus caballus*; Keeling et al., 1999), for example, by impacting the predictability of their environment or causing stress due to visual isolation from conspecifics. More generally, a better knowledge of the cognitive abilities of domestic animals could also help change how society perceives them and increase public awareness of welfare issues.

The concept of object permanence was introduced by Piaget (1954), who defined six stages of increasing complexity (Table 1). Stage 5 and below characterize the understanding of “visible displacements,” i.e., the object is visibly placed in its final location. Stage 6 corresponds to the understanding of “invisible displacement,” i.e., the object is first hidden in a displacement device and then invisibly transferred to its final location (typically inside a container or behind a screen). The displacement device is then removed and shown to be empty, enabling the subject to infer that the object has been left in this final location. Solving invisible-displacement tasks is much more demanding, as it requires mentally reconstructing the unseen displacement of the object, thereby involving secondary representations (i.e., the mental representation of an event that was not perceived directly; Suddendorf and Whiten, 2001; Jaakkola, 2014). Recently, an alternative task testing for this understanding of invisible displacement has gained attention in the field of animal cognition (for example, see Doré et al., 1996; Barth and Call, 2006; Hoffmann et al., 2011; Auersperg et al., 2014; Nawroth et al., 2015) called the transposition task. In this task, first introduced by Sophian (1985), the subject can see an object being placed into one of several opaque containers, and the containers themselves are then displaced. The subject has to track the trajectory of the hidden object. This task has the advantage of requiring considerably less memory and attention than the Piagetian task (de Blois et al., 1999; Fiset and Plourde, 2013).

**Table 1:** Description of the different stages defined by Piaget (1954).

Stage	Description
1	Unable to follow the movement of a visible object
2	Start tracking moving objects
3	Retrieve partially hidden items
4	Retrieve completely hidden items, but commit A-not-B error *A-not-B error*: if an object is repeatedly hidden in a place (A), and is then placed in another location (B), in full view of the subjects, they persevere in choosing the previously reinforced location A
5a	Retrieve an object that is hidden in a different location every time
5b	Retrieve an object that sequentially visits one or several locations before reaching its final hiding location
6a	Retrieve an object that is invisibly transferred directly from a displacement device to the final hiding place (i.e., single invisible displacement)
6b	Retrieve an object that visits one or several other hiding locations in a displacement device before being transferred to the final hiding place (i.e., double or sequential invisible displacement)

Previous results suggest a full understanding of object permanence up to Stage 6 in several non-human species, including primates (e.g., Neiworth et al., 2003; Mendes and Huber, 2004; Barth and Call, 2006), corvids (Corvidae, e.g., Bugnyar et al., 2007; Hoffmann et al., 2011), psittacids (Psittacidae, e.g., Pepperberg et al., 1997; Auersperg et al., 2014; Bastos and Taylor, 2019), pigeons (*Columba livia domestica*, Zentall and Raley, 2019), and goats (*Capra aegagrus hircus*, Nawroth et al., 2015; Vas et al., 2019). Other species succeeded in tasks involving Stage 5, but not 6. These include rhesus monkeys (*Macaca mulatta*, e.g., de Blois and Novak, 1994), prosimians (e.g., Deppe et al., 2009; Mallavarapu et al., 2013), dolphins (*Tursiops truncatus*, Jaakkola et al., 2009; Singer and Henderson, 2015), dogs (*Canis familiaris*, e.g., Collier-Baker et al., 2004; Fiset and LeBlanc, 2007, but see also Gagnon and Doré, 1992, 1993, 1994), macaws (*Ara militaris*, Wrape and Hammonds, 2019), and cats (*Felis catus*, e.g., Doré, 1986; Dumas and Doré, 1989).

The results of several studies suggest that horses (*E. caballus*) can reach Stage 5a of object permanence. For instance, they can remember the location of hidden food (McLean, 2004; Baragli et al., 2011a) and count the number of apples hidden in a bucket (Uller and Lewis, 2009). They also have a precise expectation of the identity of a conspecific once they have seen it disappear to a specific location, suggesting that they still know that individual is there even though they can no longer see it (Proops et al., 2009). However, object permanence has never been directly investigated in horses and, in particular, nothing is known about their understanding of invisible displacements. This is an important gap in our understanding of their physical cognition, especially considering its importance in terms of animal welfare. Hence, in this study, we aimed to investigate further the understanding of object permanence in horses in two experiments. In the first, we used two classical visible-displacement tasks adapted from Uzgiris and Hunt (1975) scale 1 tasks, consisting of hiding an object inside one of two and then one of three opaque containers, and a double-crossed transposition task (Sophian, 1985), testing for the horse’s understanding of invisible displacements. The performance of our subjects in this first experiment was poor; we therefore constructed new tasks, which were more straightforward for the horses in a second experiment. (1) We gave them previous experience of containment of an object, by training them to retrieve objects displaced in transparent containers before testing them in visible and invisible displacement tasks. Indeed, this previous visual experience has been suggested to enhance object permanence abilities in adult dolphins (*T. truncatus*; Pérez-Manrique and Gomilla, 2018). (2) During these training sessions, horses were also familiarized with the tasks and their purpose of choosing the baited container in order to receive the food. (3) The experimenter was totally hidden in a hut to make sure the horses focused on the task, rather than trying to use potentially confounding cues from the experimenter’s behavior as in the case of Clever Hans study of Pfungst and Rahn (1911). (4) We used a simpler single transposition task, in which only one container was displaced and the initial position of the target was left empty after the transposition.

We hypothesized that the horses would reach Stage 5a of object permanence since previous studies suggest some understanding of visible displacements in horses (e.g., Uller and Lewis, 2009; Hanggi, 2010; Baragli et al., 2011b). Nothing is known about their understanding of invisible displacements.

## Experiment 1

### Materials and Methods

#### Subjects and Husbandry

This study was conducted on 20 female adult Welsh ponies (*E. caballus*, mean age ± SE = 6.60 ± 0.79), bred at the French National Institute for Agricultural Research (INRAE, Nouzilly, France, PAO, doi: 112 10.15454/1.5573896321728955E12), were used for this study. Following a pre-test 16 horses took part in the test, one of which did not take part in the three-bucket task for practical reasons unrelated to this study (cf. 2.1.4).

During the experiment, the horses were housed indoors in groups on straw bedding. They were fed with hay and had access to water ad libitum. None of the horses had previously experienced a procedure similar to the one used in this study.

#### Ethical Note

This experiment was conducted under the authorization of the French Ministry of Agriculture (File number: 2016110908285740; this study was exempted from research ethics committee approval, as no interventions were applied to the animals). At the end of the experiment, the animals returned to normal breeding at the INRAE experimental unit. No invasive or stressful procedure was performed on them. No obvious injury or sign of pain was observed either. The horses lived in social groups and were taken to an outside paddock daily. During the experimental period, the animals were not subjected to any feed restrictions and were tested in a familiar place.

#### Procedure

**Figure 1:**
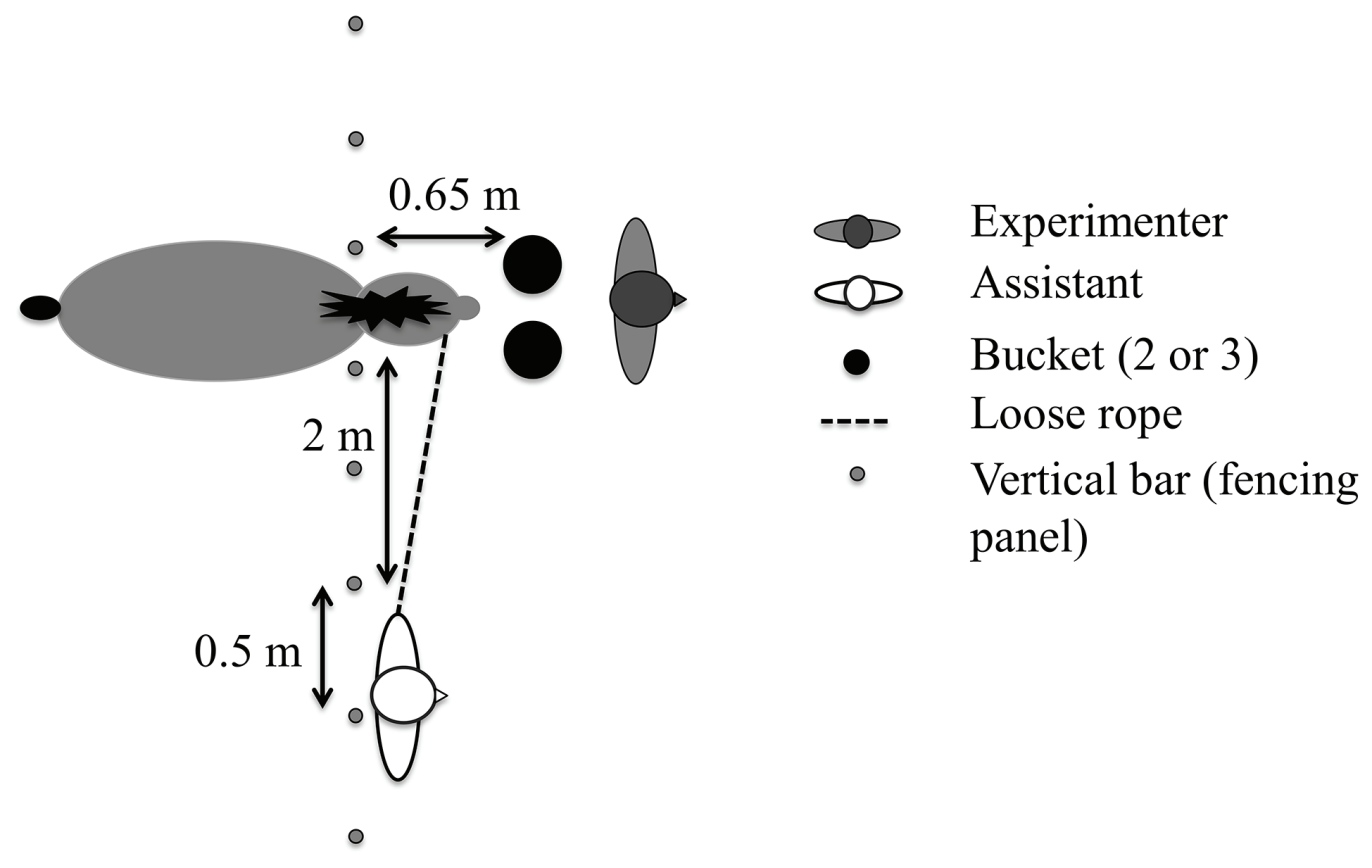
Experimental set-up. The small triangles represent the experimenter’s and the assistant’s noses: both of had their backs to the horse. All distances are approximate.

During the different tasks, an assistant held the test horse loosely on a leading rein with their back toward the horse, while an experimenter handled the bucket(s) (Figure 1). The horse stood with its head passed between the vertical bars of a fencing panel (spacing = 0.50 m) of a 12 × 12 m stall, so that it could move its head freely but could not move forward. In all tasks, food pellets were hidden alternately in one of two or three buckets in an order randomized between the horses for six trials. During the baiting, the buckets were out of the horse’s reach, approximately 1 m away; they were then closed with a lid and pushed toward the horse simultaneously. The first bucket the horse touched was defined as the chosen bucket. We considered that the horse made a choice if it touched a bucket with its nose in less than 30 s; otherwise, the trial was marked as uncompleted and a new trial started (no trial had to be restarted more than twice).

The buckets used were green plastic buckets (0.20 m in diameter and 0.10 m deep), closed by a green plastic lid. This type of bucket was unfamiliar to the horses before the beginning of this experiment. Each bucket was composed of two recipients, stacked together with food in between to create a false bottom. Horses could thus not find the target bucket by using their sense of smell, as all buckets actually contained food.

#### Tests

Before the actual test phase, we started with six pre-test trials to ensure that the horses were motivated and to familiarize them with the buckets. In the first three trials, the experimenter put food pellets in a bucket, half-closed it with a lid, and then placed the bucket on the ground approximately 0.65 m from the horse. The procedure of the three last trials was identical except that the bucket was completely closed with the lid. The horses passed a trial by directly touching the lid of the bucket within 30 s in an attempt to reach the food. The experimenter then opened the lid and let the horse eat. Only the horses that succeeded in all of the pre-test trials took part in the tests (i.e., 16 out of 20 horses).

**Figure 2:**
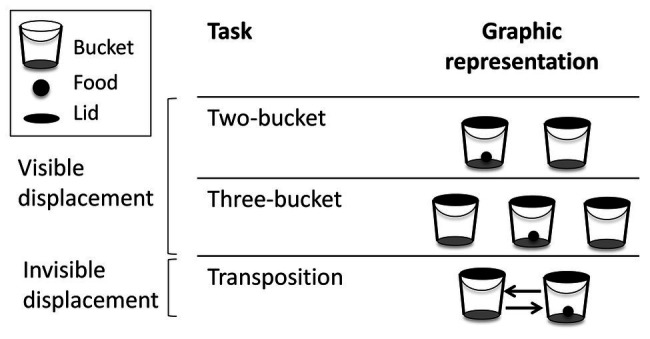
Graphic representations of the different tasks.

The different tasks are summarized in Figure 2. All tasks were videotaped using a Sony DCR-SR21E camera. The pre-tests were performed in one session, whereas the three test tasks were performed on 3 separate days to reduce the duration of the sessions so that the horses did not lose concentration or motivation for food. The two-bucket task was carried out first, then the transposition task and finally the three-bucket task. The risk of associative learning, through which the horse could learn how to solve the task by using a simple associative rule rather than object permanence (Jaakkola, 2014) was prevented by reducing the total number of trials performed and conducting each task in only one session.

1. The two-bucket task. The experimenter put the food in one of the two buckets, placed approximately 0.50 m apart at equal distance from the horse. The assistant and experimenter had their backs to the horse to prevent accidental social cueing. The experimenter interacted similarly with both buckets avoiding any attention bias of the horse toward the baited bucket by putting one hand above each bucket and then opening her hands, to drop the food pellets into one of the buckets. In addition, the two lids were closed at the same time.
2. The three-bucket task. This task was similar to the two-bucket task, except that it involved three buckets placed next to each other approximately 0.30 m apart. In this task, the experimenter had to face the horse for practical reasons, but wore a scarf on her face to hide her eyes and facial expression. Again, while placing the food pellets inside a bucket, the experimenter put her second hand above another bucket at the same time so as not to point toward one bucket in particular during the baiting. The experimenter first closed two lids and then the third one; the order in which the lids were closed was randomized. To allow the three buckets to be pushed toward the horse at the same time, they were placed on a large piece of cardboard, and the cardboard was moved.
3. The transposition task. The food pellets were again hidden in one of the two buckets (cf. two-bucket task), but then the position of the two buckets was swapped so that the left bucket was now located on the right-hand side and the right bucket on the left-hand side. Again, the experimenter had to face the horse during this task, but wore a scarf over her face. During the swapping, the right bucket always passed in front of the left bucket, independently of where the food was.

#### Statistical Analyses

The horse successfully completed a trial by touching the baited bucket first. For each horse, the number of successful trials over the six trials was calculated from the video recordings.

All statistics were performed with R 3.0.2 (R Core Team, 2013). Due to our limited sample size, we used nonparametric statistical tests. All of the tests used were two-tailed.

For each of the three tasks, we used a one-sample Wilcoxon signed-rank test to determine whether, at the group level, the proportion of successful trials obtained by each individual over the six trials differed from chance level (fixed at 0.50 for the two-bucket and the transposition tasks and at 0.33 for the three-bucket task).

### Results

All the results from the different tasks are summarized in Table 2.

**Table 2:** Performance of each horse in the different tasks. Results are given as the number of successful trials over the total number of trials.

Individuals	Number of successful trials/total number of trials		
	Two-bucket	Three-bucket	Transposition
Flavie	4/6	2/6	4/6
Fantasy	4/6	3/6	3/6
Filharmonie	3/6	2/6	3/6
Furibonde	3/6	4/6	3/6
Forever	5/6	2/6	4/6
Elegante	5/6	4/6	3/6
Estampe	5/6	4/6	3/6
Eloge	3/6	2/6	4/6
Eclipse	4/6	2/6	3/6
Dolly	2/6	4/6	1/6
Diva	2/6	1/6	4/6
Danseuse	3/6	X	2/6
Cybele	3/6	3/6	3/6
Berenice	6/6	4/6	5/6
Valentine	3/6	3/6	3/6
Victoria	5/6	4/6	6/6
*N*	16	15	16
Mean proportion of success	0.62^*^	0.49^*^	0.56^NS^
Chance-level	0.50	0.33	0.33

“X” indicates that the horse could not be tested for practical reasons. Significance was assessed by a Wilcoxon signed-rank:

NS *p* > 0.05;

* *p* ≤ 0.05.

#### Two-Bucket and Three-Bucket Tasks

Horses chose the correct bucket more often than expected by chance when tested with two buckets [*N* = 16, *M* = 0.62, 95% CI (0.50, 0.83), *V* = 52, *p* = 0.013] and with three buckets [*N* = 15, *M* = 0.49, 95% CI (0.33, 0.67), *V* = 54, *p* = 0.006].

#### Transposition Task

Horses did not choose the baited bucket significantly more often than expected by chance [*N* = 16, *M* = 0.56, 95% CI (0.50, 0.67), *V* = 28.5, *p* = 0.156)]

### Summary

In the two-bucket and the three-bucket tasks, horses were able to retrieve an object (here, food) that had been hidden in two or three different locations in several successive trials, reaching Stage 5a of object permanence. However, even though they chose the baited bucket significantly more often than expected by chance as a group, the mean proportions of success of the horses were not very high. Moreover, horses failed to choose the baited bucket more often than expected by chance in the transposition task. Hence, our results do not support that horses can reach Stage 6a.

Their poor performances in these tasks could be explained by different factors. (1) Our subjects could lack experience with hidden objects, as they might not have had a lot of opportunities to interact with hidden objects in their life. Indeed, in infants and chicks it has been suggested that such interactions can be important for the development of object permanence (Bremner et al., 2015; Prasad et al., 2019). Giving the horses opportunities to interact with hidden objects prior to testing them in the different tasks might increase their performances, as has been shown in adult dolphins for instance (*T. truncatus*; Pérez-Manrique and Gomilla, 2018). (2) Horses might have not been familiar with the purpose of the task, namely that they had to choose only one bucket and that if they chose the baited bucket they could eat the food inside. (3) Instead, horses might have sought to use a rule based on the experimenter’s behavior, as in the case of Clever Hans (Pfungst and Rahn, 1911). As the experimenter took the utmost care not to indicate the baited bucket, the arbitrary social cues horses could use might have been confounding and could have hinder their performance in the task. (4) The crossed-transposition task can be confounding for some species (e.g., in dogs, *C. familiaris*; Miller et al., 2009) because the two containers are displaced at the same time, which can be distracting for the subject, and because there is a lack of visual change between the final situation after transposition and the initial situation prior to transposition.

Hence, we carried out a second experiment with different horses and a different protocol in an attempt to address these potential issues. (1–2) Horses were first trained to retrieve a food reward hidden in a transparent container, before testing them in visible and invisible displacement tasks. (3) The experimenter was completely hidden from the horse in a hut. (4) We used a single transposition task: only one cup was displaced at a time and the initial position of the target was left empty after the transposition.

## Experiment 2

### Materials and Methods

#### Subjects and Husbandry

This experiment was conducted on 14 other female Welsh ponies from 6 to 9 years old (mean age ± SE = 7.90 ± 0.31). From these 14 horses, 10 were used in the two-cup test and six in the three-cup test because the other horses did not reach the success criterion during the training sessions. For practical reasons, the experiment had a limited time period in which to conduct the test, and therefore only three horses (Ukraine, Uppsala, and Ballerine) finished the three-cup test quickly enough to be trained and tested in the single-transposition (ST) test.

#### Apparatus and General Procedure

**Figure 3:**
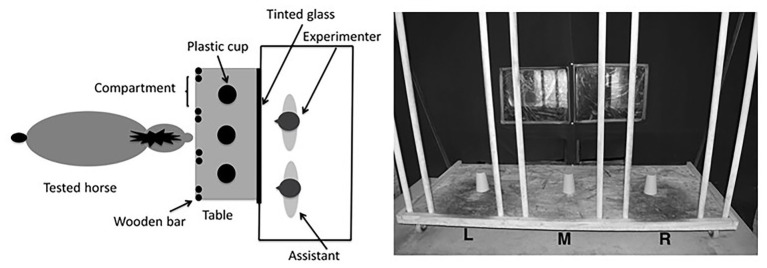
Arrangement of the experimental apparatus. On the left, a schematic representation shows how the horse and the experimenters were located around the apparatus. The small triangles represent the experimenter’s and the assistant’s noses: both of them faced the horse. On the right, a picture shows the sliding tray, divided into three compartments by wooden bars (L, M, and R). The three plastic cups are arranged as they were for the three-cup test.

The apparatus consisted of a sliding wooden tray (1.40 × 0.60 m), placed on a table (1.80 ml × 1.20 mw × 0.57 mh; cf. Figure 3). The tray could be slid by the experimenter from an adjacent hut, using a handle (0.84 m long). The experimenter and the assistant were hidden in this hut (2.30 × 1.07 × 1.86 m) during the entire duration of the test session in order to avoid social cueing. Both could see the horse through two tinted-glass windows and the experimenter could pass her hands through two small holes (0.27 × 0.15 m, hidden by black fabric) to place and manipulate the experimental material on the tray. The tray was divided into three compartments (each 0.28 m wide) by four pairs of wooden bars: one on the left (compartment L), one in the middle (compartment M), and one on the right (compartment R).

During the experiment, the horse was loosely attached with a leading rein in front of the apparatus. The experimenter first attracted the horse’s attention by knocking on the window of the hut. In full view of the horse, a food reward was placed by the experimenter into one of the three compartments (approximately 1.00 m away from the horse) and two or three green, opaque, plastic cups (0.06 m in diameter and 0.09 m high) were placed on the tray, one per compartment and with one of them hiding the reward. Prior to this experiment, the horses were unfamiliar with these cups. We chose to use cups instead of buckets, as cups were easier for the experimenter to handle from inside the hut. A food reward was stuck at the bottom of each cup, to control for sensory cues. The experimenter then pushed the sliding tray toward the horse, so that the horse could pass its head between the wooden bars, into the appropriate compartment, knock over the plastic cup and eat the reward. The test and training sessions consisted of several of these trials in a row: the location of the reward during each trial was semi-randomized, so that it was never hidden in the same compartment more than twice in a row. The risk of associative learning was reduced by interacting similarly with the different cups.

The horses were tested individually, 5 days a week, typically twice a day during 8–10-min sessions (either test sessions or training sessions). Four different test types were carried out as described below.

#### Training

Each test was preceded by a training period, including specific training for this test type and basic training. This training aimed at providing horses with visual experience of hidden objects. Moreover, during the specific training, horses learned to choose one and only one cup to that they had to find the food reward in order to be allowed to eat it. Basic training served to familiarize the horses with the apparatus (the sliding tray and the plastic cups) and then as a means to ensure motivation. The sessions were started with the simple basic training task followed by the more complex specific training task.

Our success criterion to enable a horse to start taking a test was to succeed in the corresponding specific training task for four consecutive sessions over 2 days or for at least five sessions over 3 days.

1. Basic training: find-the-reward training (FR). The reward was placed in one of the compartments and stayed in full view of the horse. The horses received either three trials – if they succeeded in all three – or six trials. They were considered to have succeeded in this task if they passed their head into the baited compartment at the first attempt, either for the first three or for five out of six trials.
2. Basic training: one-cup training (1C). In full view of the horse, the reward was placed into one of the compartments and was then hidden under a single green plastic cup. The number of trials and the success criterion were the same as in the FR training.
3. Specific training: two-transparent-cup (2TC), three-transparent-cup (3TC), and single-transposition training. The general procedure used during these training sessions was exactly the same as the one used during the corresponding tests except that the cups used were transparent. Horses succeeded in this task if they chose the baited cup for five out of six trials.

Horses reached success criterion after 71.1 ± 7.5 (Mean ± SEM) training sessions (corresponding to 35.5 ± 3.7 days) for the two-cup test; after 21.7 ± 9.1 sessions (or 10.8 ± 4.5 days), for the three-cup test; after 28.7 ± 9.7 sessions (or 14.3 ± 4.8 days) for the single-transposition test.

#### Tests

**Figure 4:**
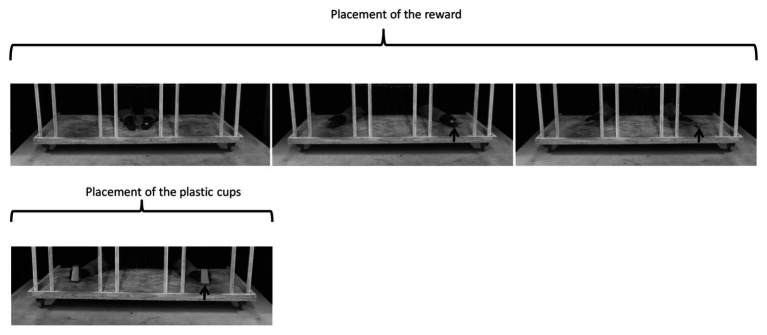
Detailed procedure used for placing the reward and plastic cups during the two-cup test. The outlines of the reward and of the cups have been highlighted to improve their visibility in the pictures. The arrow indicates the location of the reward.

**Figure 5:**
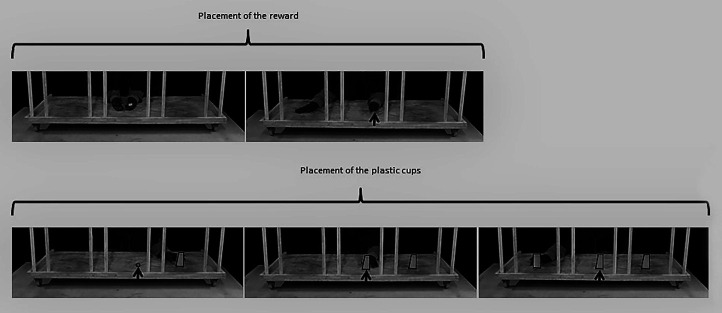
Detailed procedure used for the placing the reward and plastic cups during the three-cup test. The outlines of the reward and the cups have been highlighted to improve their visibility in the pictures. The arrow indicates the location of the reward.

**Figure 6:**
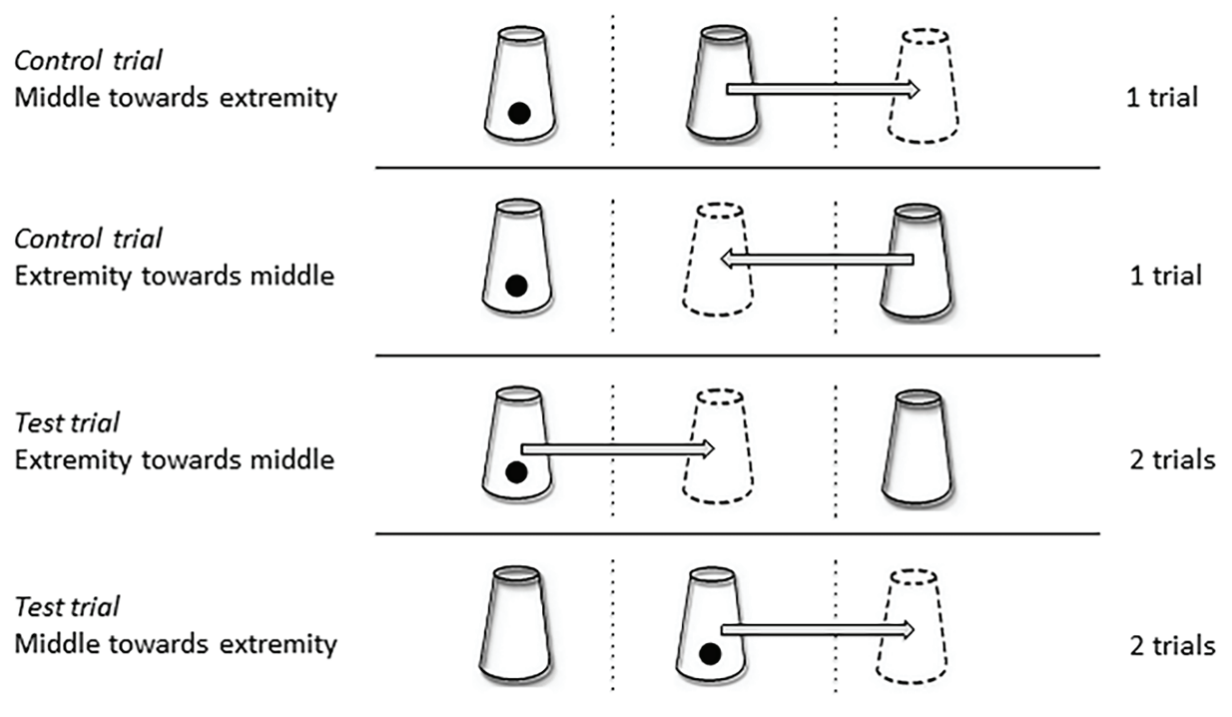
Schematic representation of the four types of trials. The cups drawn in dotted lines represent the final positions of the cup, after transposition; the arrows represent the movement of the cups during the transposition. In “Extremity towards middle” trials, the cups were shifted from the middle to an extremity, while for the “Middle towards extremity” trials the cups were shifted from an extremity to the middle. These four types of trials were randomized during each test session (the number of trials conducted for each type is indicated on the right).

Three different types of test were performed (Figures 4–6).

1. The two-cup test: a food reward was hidden under one of two opaque plastic cups (one in compartment L and the other in compartment R).
2. The three-cup-test: a food reward was hidden under one of three opaque plastic cups (one cup in each compartment).
3. The single-transposition test: a food reward was hidden under one of two opaque plastic cups (placed in two of the three compartments) and one of the cups was then moved to an adjacent compartment.

All the tests were performed over six consecutive sessions, consisting of six trials each, over 3 days. At the beginning of each test session, pre-test training trials were performed in order to ensure horses were attentive and motivated. These pre-test training trials consisted of three or six trials of the 1C and three to six trials of the specific training corresponding to the test (2TC, 3TC, or ST). If the horse failed the pre-test training three times during a session, the session was interrupted and a supplementary test session was added at the end of the 3 days. If more than one test session had to be interrupted in that way, it was considered that the horse did not sufficiently master the training tasks and additional training sessions were added until the horse met the success criterion again.

During the test (and training) trials, the way the food reward and the plastic cups were positioned on the sliding tray was highly standardized and was designed to be symmetrical in order to prevent associative learning.

1. Two-cup test (Figure 4):
  - Placing the reward: the experimenter extended her two hands out through the holes in the hut, one of them holding the reward, and showed their content to the horse by opening her hands palm upward, in the middle compartment of the tray. The two open hands (with the reward visible) were then moved simultaneously and symmetrically: the left hand to compartment R and the right hand to compartment L. The experimenter put the reward into the target compartment, and took her hands back inside the hut.
  - Placing the plastic cups: the experimenter took two opaque plastic cups out, one in each hand, and placed them simultaneously and symmetrically in the two compartments, one of them covering the reward. The experimenter then took her hands back inside the hut.
2. Three-cup-test sessions (Figure 5):
  - Placing the reward: the reward was placed in exactly the same way as in the two-cup test except that, after extending her two hands palm upward in the middle compartment of the tray, the experimenter moved her hands simultaneously toward two of the three compartments used (the baited compartment and one of the other two which was assigned randomly). The hand holding the reward placed it into the baited compartment, while the empty hand performed exactly the same gesture in another compartment. The purpose of this procedure was to avoid interacting with the baited compartment only, to prevent the horses forming associative rules.
  - Placing the plastic cups: the three cups were placed successively from the right to the left of the horse. The experimenter took the cups out one at a time: she placed the first one into compartment R with her left hand and the two others into compartments M and L with her right hand. She then took her hands back inside the hut.
3. Single-transposition test (Figure 6):
  - Placing the reward: the reward was placed in one of the three compartments, using the same procedure as described previously for the three-cup test.
  - Placing the plastic cups: the experimenter took two plastic cups out, one in each hand, and placed them simultaneously in two out of the three compartments (randomly assigned), one of them covering the reward.
  - Displacing the plastic cups: the experimenter shifted one of the plastic cups to an adjacent compartment (so either from the middle – compartment M – toward an extremity ‐ compartment L/R, or from an extremity toward the middle). During this procedure, the experimenter kept the other hand on the motionless cup in order to act in a similar way with the two cups.

Two types of trials were carried out. (1) In the control trials, the displaced cup was empty, while (2) in the test trials, it was the baited cup. Each test session consisted of two control trials (one in which the cup was shifted from the middle to an extremity and the other from an extremity toward the middle) and four test trials (two of each type), carried out in a random order.

#### Statistical Analyses

Horses were considered to have chosen a specific compartment if they moved their head into this compartment first and fully extended their head between the wooden bars of this compartment.

Horse performances at group and individual levels were assessed through the following procedures:

1. Two-cup and three-cup tests: the number of successfully completed trials for the entire test (i.e., combining all the sessions, over the 36 trials) was used as a dependent measure for each test.
2. Single-transposition test: the number of successfully completed trials for the test trials of the entire test (i.e., over 24 trials) and the number of successfully completed trials for the control trials of the entire test (i.e., over 12 trials) were used as a dependent measures. Due to the limited sample size (*N* = 3), we could not perform a Wilcoxon signed-rank test at the group level. Therefore, we only tested significance at the individual level. Moreover, for the horses that significantly succeeded in both test and control trials, binomial tests were also carried out to investigate whether they succeeded at an above chance level when considering the extremity-toward-middle trials only or the middle-toward-extremity trials only.

Exact binomial tests were run to compare the individual performance of each horse with chance level (fixed at 0.50 for the two-cup and single-transposition tests and 0.33 for the three-cup test). We used a Wilcoxon signed-rank test to compare the proportion of success of the whole group with chance level.

### Results

#### Two-Cup and Three-Cup Tests

**Figure 7:**
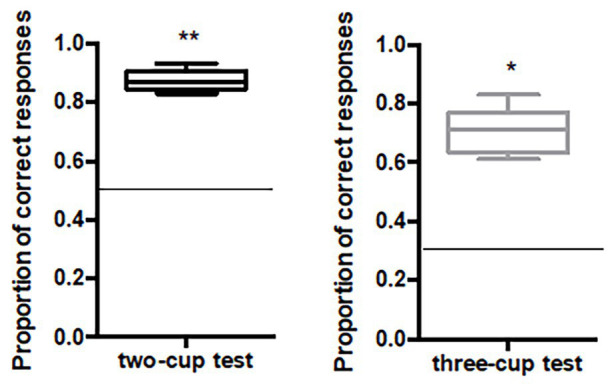
Median proportion of correct responses for the two-cup and the three-cup test. Horizontal lines indicate chance levels (fixed at 0.5 for the two-cup test and at 0.33 for the three-cup test). Deviation from chance was assessed by a Wilcoxon signed-rank test. ^*^p ≤ 0.05, ^**^p < 0.01.

When considered at group level, horses performed significantly above the chance level for the two-cup test [*N* = 10, *M* = 0.87, 95% CI (0.84, 0.91), *V* = 55, *p* = 0.006] and the three-cup test [*N* = 6, *M* = 0.70, 95% CI (0.67, 0.71), *V* = 21, *p* = 0.035; Figure 7]. Moreover, all of the horses individually scored significantly higher than expected by chance both in the two‐ and the three-cup tests (Table 3).

**Table 3:** Proportion of correct responses for each test, for each individual. Deviation from chance at the individual level was tested by an exact binomial test.

Individuals	Number of successful trials over the total number of trials			
	2-cup test	3-cup test	S.t. test trials	S.t. control trials
Ukraine	33/36^***^	25/36^***^	23/24^***^	9/12^NS^
Uppsala	32/36^***^	32/36^***^	18/24^*^	12/12^***^
Ballerine	35/36^***^	26/36^***^	16/24^NS^	9/12^NS^
Valda	31/36^***^	25/36^***^		
Volga	30/36^***^	21/36^**^		
Aventure	30/36^***^	24/36^***^		
Urielle	34/36^***^	25/36^***^		
Vanille	31/36^***^			
Vaillante	28/36^**^			
Altesse	31/36^***^			
Mean proportion of success	0.87^**^	0.70^*^	0.79	0.83
Total number of trials	36	36	24	12
Chance level	0.50	0.33	0.50	0.50

Deviation from chance at the group level was tested by a Wilcoxon signed rank test. S.t stands for “single-transposition (ST).”

NS *p* > 0.05;

* *p* ≤ 0.05;

** *p* < 0.01;

*** *p* < 0.001.

#### Single-Transposition Test

The three horses reached the success criterion during training sessions and thus took the single-transposition test. Two of them (Ukraine and Uppsala) performed significantly better than expected by chance in the single-transposition test trials. Only Uppsala succeeded significantly better than expected by chance in the single-transposition control trials (Table 3).

Further binomial tests showed that Uppsala succeeded significantly more often than expected by chance in the extremity-toward-middle trials [success rate = 0.92, 95% CI (0.61, 1.00), *p* = 0.006] but not in the middle-toward-extremity trials [success rate = 0.58, 95% CI (0.28, 0.85), *p* = 0.774].

### Summary

All of the tested subjects succeeded in visible-displacement tasks (Stage 5a), whether they were tested with two or three possible hiding places.

In the invisible-displacement task (Stage 6a), two out of three horses succeeded in the test, but one of them failed in the control trials, which suggests that it relied on an associative rule (i.e., always choosing the cup that was displaced), rather than understanding visible displacement. The horse (Uppsala) that succeeded in both the test and the control trials did not exceed chance level when the baited cup was moved from the middle compartment toward an extremity. This result suggests that this horse might have used an associative rule as well. Fiset and Plourde (2013), who performed a similar experiment in dogs (*C. familiaris*) and wolves (*Canis lupus*), suggested that they might have succeeded in this task by selecting the container closest to the newly empty baited position instead of solving invisible displacements. This hypothesis could explain the results of Uppsala as well: the middle-toward-extremity trials might have been more difficult for it since both cups were equidistant to the newly empty previously baited compartment. Hence, although the results from this transposition test should be considered with caution because of the low sample size, they do not support the hypothesis that horses comprehend invisible displacement. Nevertheless, most of the horses tested could still use alternative strategies (probably learned by associative learning) to solve the task, which demonstrates their flexibility.

Interestingly, the horses obtained higher proportions of success in the Stage 5a tasks of experiment 2 (two-cup test = 87%; three-cup test = 70%) than in experiment 1 (two-bucket task = 62%; three-bucket task = 49%). Since a previous study shows that horses perform better in visual discrimination tasks when the stimuli were on the ground than at nose level (Hall et al., 2003), we could have expected that horses would have performed better in experiment 1 (when the buckets were on the ground) than in experiment 2 (when the cups were on a table), but this was not the case.

The better results in experiment 2 could be explained by the adjustments made in this experiment compared to experiment 1, and in particular due to the additional training phase, during which horses were trained to retrieve objects placed in transparent containers. This training could have helped the horses to gain more experience with hidden objects. The importance of such previous experience has been shown in dolphins (*T. truncatus*) for instance (Pérez-Manrique and Gomilla, 2018). Moreover, in these training sessions, horses could learn to understand the purpose of the tasks, namely that they had to choose only one cup and to find the food reward in order to be allowed to eat it. The training could also have improved their working memory and attention span. Furthermore, it could be related to the fact that the experimenter was totally hidden in a hut during the experiment 2. This prevented the horses from seeking to use potentially confounding cues from the experimenter’s behavior (as in the case of Clever Hans, Pfungst and Rahn, 1911). Alternatively, the large number of training sessions in experiment 2 might have resulted in associative learning: horses could have learned associative rules during the training to find the reward more accurately than in experiment 1, in which they could only rely on their object permanence abilities. We used transparent cups during the training to reduce this possibility: horses could see the content of the cups at all times and thus did not need to develop associative rules to find the food reward. Nevertheless, this cannot be excluded, particularly since the results of the transposition test suggest that they used associative rules.

## General Discussion

We carried out two experiments with different horses. In the first experiment, we used two classic paradigms to test for Stages 5a and 6a of object permanence. In the second experiment, we tested these two same stages with a different protocol designed to make the tasks easier for the horses by avoiding potentially confounding factors. In both experiments, horses were successful in the visible-displacement tasks, whether they were tested with two or three possible hiding places. These results suggest that adult horses can comprehend visible displacement and hence reach Stage 5a of object permanence following the Piagetian framework. However, our horses did not succeed in the transposition tasks, either in experiment 1, or in the simpler version used in experiment 2. Hence, our results do not support that horses understand invisible displacements (Stage 6a of object permanence).

Special care was taken in both experiments to avoid bias due to sensory or social cueing and to limit the risk of associative learning, although the latter cannot be totally excluded in experiment 2, as it involved a large number of training sessions.

### Horses Solved All Visible-Displacements Tasks (Stage 5a)

In both experiments, horses succeeded in the tasks involving visible displacement. They were able to retrieve an object that was hidden in two or three different locations in several successive trials (Stage 5a). Our study is thus the first to show directly that horses can understand visible displacement, suggested in several previous studies. For instance, horses were found to be able to remember the location of hidden food (McLean, 2004; Baragli et al., 2011a); to count the number of apples hidden in a bucket (Uller and Lewis, 2009); to ask their caretaker for help to reach a hidden food source (Ringhofer and Yamamoto, 2016; Trösch et al., 2019); and to form a precise expectation of the identity of a conspecific once they saw it disappear to a specific location (Proops et al., 2009). Further studies, testing whether horses can successfully complete a sequential visible-displacement task (Stage 5b) would be interesting to complement our results.

### Horses Failed to Solve the Invisible-Displacement Tasks (Stage 6a)

In experiment 1, horses failed to choose the baited container more often than expected by chance in the transposition test. In the single-transposition test of experiment 2 (Stage 6a), some horses succeeded, but seem to have relied on associative rules rather than on an understanding of invisible displacements. As the Piagetian framework and the different tasks used in this study were originally established for humans, we could consider whether horses failed these tasks because of limited object permanence ability or because these tasks are not adapted to horses (Pepperberg, 2002). For instance, we could wonder if horses failed because of an insufficient short-term memory, as transposition tasks require higher short-term memory capacities than visible-displacement tasks since the time between the moment the food item is hidden and the moment the horse can start searching for it is longer due to the time taken for the containers to be displaced (Jaakkola et al., 2009). However, this time period was only approximately 5 s in our study, which is greatly inferior to the working memory duration of 20s reported for horses by Valenchon et al. (2013), who used a similar protocol. Furthermore, as our horses were successful in all of the visible-displacement tasks, the ecological relevance of our experimental set-up or the motivation to find food is unlikely to have been a source of failure. It would nevertheless be interesting to study further how horses understand invisible displacement using a more naturalistic set-up (for instance in the context of the disappearance of a human or a conspecific rather than food). In view of our current results, we thus cannot conclude that horses comprehend invisible displacements. Similar results of failing to comprehend invisible displacements have been found in other species, including dolphins (*T. truncatus*; Jaakkola et al., 2009), dogs (*C. familiaris*; Collier-Baker et al., 2004; Fiset and LeBlanc, 2007), rhesus monkeys (*M. mulatta*; de Blois and Novak, 1994), prosimians (Deppe et al., 2009; Mallavarapu et al., 2013), and cats (*F. catus*; Doré, 1986; Dumas and Doré, 1989). It has been argued that most invisible displacements in natural conditions could actually be partly solved by a reliance on external cues; for example, moving objects or subjects – food items, predators, or conspecifics – can also be detected through the use of other sensory cues, and usually reappear near to where they were last seen by the individual (Jaakkola, 2014). Nevertheless, horses in our study showed that they could flexibly use associative rules to deal with this type of displacements. It is possible that horses are able to comprehend invisible displacements but only rely on this more complex strategy as “a last resort,” when there is no easier alternative strategy available. In our experiment, they still received rewards regularly by using simpler associative rules, which might explain why they did not use their ability to understand invisible displacement.

In both experiments, the containers were placed 1 m from the subject during the transposition trial, as horses have been shown to have low visual acuity at distances under 1 m. Nevertheless, in both experiments, there were bars forming a barrier between the subject and the containers, which has been shown to reduce dogs’ performances in an object choice task. This phenomenon might also be true for horses and could have decreased their performances. Hence, it might be interesting for further studies to design a different protocol that does not involve a barrier.

### Conclusion and Implications for Animal Welfare

To conclude, the results from our two experiments, using different horses and protocols show that horses seem to understand visible displacements. Horses thus reach Stage 5a of object permanence following the Piagetian framework. However, horses failed the transposition tasks, suggesting potential lack of understanding of invisible displacements (Stage 6a).

When considered in a more applied context, our results highlight that horses’ perception of the external world, and in particular their reasoning about invisible displacements, differs considerably from our own. Horses seemed to fail in a transposition task that is easily solved by human adults. It is particularly important to take this new information into account as current husbandry methods usually involve walls and opaque barriers that limit the field of view of horses (Nawroth et al., 2019). This incomplete understanding of object permanence might thus increase the unpredictability of their world. For instance, horses might have difficulties in anticipating the re-appearance of objects or individuals that were out-of-sight (Désiré et al., 2002), potentially inducing stress (example in lambs: Désiré et al., 2004). Moreover, being visually isolated from conspecifics could also result in a stressful situation since horses are gregarious animals (Waring, 2003). Addressing these potential stressors in horse husbandry and equestrian practices, could contribute to improving their well-being and reducing the occurrence of horse-related accidents, whose major cause is fear reactions (Keeling et al., 1999).

## Data Availability Statement

The raw data supporting the conclusions of this article will be made available by the authors, without undue reservation.

## Ethics Statement

Ethical review and approval was not required for the animal study because no interventions were applied to the animals (Val de Loire Ethical Committee, CEEA, VDL, France; file number: 2016110908285740).

## Author Contributions

MT and LL: conceptualization. MT and LL: methodology. LL: validation. MT, AF, and LL: formal analysis. MT, AF, and MC: investigation. LL: resources. MT and AF: data curation. MT: writing – original draft preparation. MT, LL, RN, and LC: writing – review and editing. MT, LL, RN, and LC: visualization. LL: supervision, project administration and funding acquisition. All authors contributed to the article and approved the submitted version.

### Conflict of Interest

The authors declare that the research was conducted in the absence of any commercial or financial relationships that could be construed as a potential conflict of interest.
